# Supplementary material for: HIVprotI: an integrated web based platform for prediction and design of HIV proteins inhibitors
Source: J Cheminform. 2018 Mar 9;10:12. doi: 10.1186/s13321-018-0266-y (PMC5845081; doi:10.1186/s13321-018-0266-y)
Supplement: Supplementary file 2 — Additional file 2. Source code of HIVProtI web server. [file 13321_2018_266_MOESM2_ESM.zip › HIVprotI_Source-code/hivprotI/weka-3-6-6/doc/weka/gui/beans/InstanceEvent.html]

InstanceEvent


---


|  |  |  |
| --- | --- | --- |
| **Overview** \| **Package** \| **Class** \| **Tree** \| **Deprecated** \| **Index** \| **Help** \| **Weka's home** | |  |
| **PREV CLASS**   **NEXT CLASS** | **FRAMES**    **NO FRAMES**     **All Classes** |  |
| SUMMARY: NESTED \| FIELD \| CONSTR \| METHOD | DETAIL: FIELD \| CONSTR \| METHOD |  |


---


## weka.gui.beans Class InstanceEvent

```
java.lang.Object
  java.util.EventObject
      weka.gui.beans.InstanceEvent
```

**All Implemented Interfaces:**: java.io.Serializable

---

``` public class InstanceEvent extends java.util.EventObject ```

Event that encapsulates a single instance or header information only

**Version:**
:   $Revision: 1.5 $

**Author:**
:   Mark Hall

**See Also:**: `EventObject`, Serialized Form

---

| **Field Summary** | |
| --- | --- |
| `static int` | `BATCH_FINISHED` |
| `static int` | `FORMAT_AVAILABLE` |
| `static int` | `INSTANCE_AVAILABLE` |


**Constructor Summary**

`InstanceEvent(java.lang.Object source)`

`InstanceEvent(java.lang.Object source,
Instance instance,
int status)`
  
          Creates a new `InstanceEvent` instance that encapsulates
a single instance only.

`InstanceEvent(java.lang.Object source,
Instances structure)`
  
          Creates a new `InstanceEvent` instance which encapsulates
header information only.


| **Method Summary** | |
| --- | --- |
| `Instance` | `getInstance()`             Get the instance |
| `int` | `getStatus()`             Get the status |
| `Instances` | `getStructure()`             Get the instances structure (may be null if this is not a FORMAT\_AVAILABLE event) |
| `void` | `setInstance(Instance i)`             Set the instance |
| `void` | `setStatus(int s)`             Set the status |
| `void` | `setStructure(Instances structure)`             Set the instances structure |

**Methods inherited from class java.util.EventObject**

`getSource, toString`

**Methods inherited from class java.lang.Object**

`equals, getClass, hashCode, notify, notifyAll, wait, wait, wait`

**Field Detail**

### FORMAT\_AVAILABLE

```
public static final int FORMAT_AVAILABLE
```

**See Also:**: Constant Field Values

---


### INSTANCE\_AVAILABLE

```
public static final int INSTANCE_AVAILABLE
```

**See Also:**: Constant Field Values

---


### BATCH\_FINISHED

```
public static final int BATCH_FINISHED
```

**See Also:**: Constant Field Values


**Constructor Detail**

### InstanceEvent

```
public InstanceEvent(java.lang.Object source,
                     Instance instance,
                     int status)
```

:   Creates a new `InstanceEvent` instance that encapsulates
    a single instance only.

    **Parameters:**: `source` - the source of the event: `instance` - the instance: `status` - status code (either INSTANCE\_AVAILABLE or BATCH\_FINISHED)

---


### InstanceEvent

```
public InstanceEvent(java.lang.Object source,
                     Instances structure)
```

:   Creates a new `InstanceEvent` instance which encapsulates
    header information only.

    **Parameters:**: `source` - an `Object` value: `structure` - an `Instances` value

---


### InstanceEvent

```
public InstanceEvent(java.lang.Object source)
```


**Method Detail**

### getInstance

```
public Instance getInstance()
```

:   Get the instance

    :   **Returns:**: an `Instance` value

---


### setInstance

```
public void setInstance(Instance i)
```

:   Set the instance

    :   **Parameters:**: `i` - an `Instance` value

---


### getStatus

```
public int getStatus()
```

:   Get the status

    :   **Returns:**: an `int` value

---


### setStatus

```
public void setStatus(int s)
```

:   Set the status

    :   **Parameters:**: `s` - an `int` value

---


### setStructure

```
public void setStructure(Instances structure)
```

:   Set the instances structure

    :   **Parameters:**: `structure` - an `Instances` value

---


### getStructure

```
public Instances getStructure()
```

:   Get the instances structure (may be null if this is not
    a FORMAT\_AVAILABLE event)

    :   **Returns:**: an `Instances` value


---


|  |  |  |
| --- | --- | --- |
| **Overview** \| **Package** \| **Class** \| **Tree** \| **Deprecated** \| **Index** \| **Help** \| **Weka's home** | |  |
| **PREV CLASS**   **NEXT CLASS** | **FRAMES**    **NO FRAMES**     **All Classes** |  |
| SUMMARY: NESTED \| FIELD \| CONSTR \| METHOD | DETAIL: FIELD \| CONSTR \| METHOD |  |


---
